# An ecosystems approach for health-enhancing physical activity promotion: introducing the 11th Conference of HEPA Europe

**DOI:** 10.1093/eurpub/ckac084

**Published:** 2022-08-26

**Authors:** Viktoria A Kovacs, Anne Vuillemin, Marie H Murphy, Kremlin Wickramasinghe, Stephen Whiting, Adriana Pinedo, Harry Rutter

**Affiliations:** WHO Regional Office for Europe, Copenhagen, Denmark; LAMHESS, Université Côte d’Azur, Nice, France; UIster University Doctoral College, Newtownabbey, Northern Ireland, UK; WHO Regional Office for Europe, Copenhagen, Denmark; WHO Regional Office for Europe, Copenhagen, Denmark; WHO Regional Office for Europe, Copenhagen, Denmark; Department of Social and Policy Sciences, University of Bath, Bath, UK

Physical inactivity is responsible for over 5 million premature deaths every year. Around 20% of adults and 80% of adolescents are insufficiently physically active, women are less active than men, and inactivity is higher in older age groups and in high-income countries.^1^ There has been clear evidence for the benefits of physical activity for health for decades, but few countries have been successful at increasing population levels of activity, and physical inactivity is still a leading risk factor for premature mortality worldwide. Achieving a breakthrough requires a systems approach that combines individual-level behaviour change approaches with upstream policy actions to focus on populations, the drivers of physical activity and inactivity and the interactions between them across complex systems.^2^

This supplement has been prompted by and published ahead of the 11th Conference of HEPA EUROPE and builds on its mission to provide a forum for the advancement of health-enhancing physical activity (HEPA) research, policy and practice for better health and well-being across the WHO European Region. It seeks to strengthen the systems approach to HEPA promotion by presenting a collection of papers addressing a range of domains of physical activity including transport, workplace, school and leisure.

An appreciation of the wider determinants of physical activity and the interaction between them could help drive more effective action to promote physical activity. This is exemplified by the WHO Global Action Plan on Physical Activity (GAPPA), which features four objectives, the creation of active societies, active environments, active people and active systems.^3^ In this supplement, Power *et al.* provide an example of how a systems approach using the GAPPA map combined with social network analysis could be applied in the national-level walking promotion. The systems mapping approach catalysed cross-sectoral communication and allowed stakeholders to jointly identify several actions to drive change.

Physical inactivity reached epidemic proportions even before the coronavirus disease 2019 (COVID-19) pandemic hit the world, and the global emergency along with the introduced countermeasures further worsened the situation.^4^ Unless societies act urgently to address the epidemic of physical inactivity, they will entrench huge and growing health and economic costs into the future. The study by Racine *et al.* in this supplement estimates that in France alone sedentary behaviour causes 50 000 premature deaths, costs almost €500 million in health care costs, and around another €500 million in productivity loss every year.

Instead of working in silos, researchers, policymakers and practitioners must collaborate with each other and with other sectors, and step up to advocate loudly about the consequences of failing to increase HEPA. In an effort to establish cross-border partnerships for physical activity promotion, Tcymbal *et al*. outline in this special issue the development of the European Union (EU) Physical Activity Focal Points Network, evaluate its outputs and benefits and describe its potential and challenges. They found that the network facilitated knowledge and experience sharing in physical activity promotion at the national level. By harmonizing data collection, it has contributed to the monitoring of the implementation of European strategies and enabled comparison of implementation between the EU Member States.

In countries such as Portugal, where the share of adults with diabetes is the second highest in Europe,^5^ the need for bolder intervention is urgent. To support a decision on the large-scale introduction of physical activity interventions in diabetes management, economic evaluation is one of the important steps. In their systematic review, Barbosa *et al.* report that physical activity interventions are cost-effective in type 2 diabetes management, even cost-saving in some cases. Such findings are important for driving investments to increase physical activity.

If current trends continue, the 2025 global physical activity target of a 10% relative reduction in insufficient physical activity will not be met. A multi-sectoral systems approach to increase physical activity must urgently be prioritized. While the COVID-19 pandemic is undoubtedly one of the greatest crises of our history it has also created opportunities to build back better, fairer and more sustainably. The pandemic brought together multiple stakeholders and sectors, and countries must seize the opportunity they now have to create healthier, more active societies.
